# Nafamostat mesylate augments survival in rats afflicted by exertional heat stroke

**DOI:** 10.3389/fphar.2025.1559181

**Published:** 2025-05-15

**Authors:** Qingwei Lin, Zhuqing Luo, Longping He, Lincui Zhong, Qingbo Zeng, Ye Zhou, Qi Chen, Xingping Deng, Xiaomin Song, Qing Song, Jingchun Song

**Affiliations:** ^1^ Intensive Care Unit, The 908th Hospital of Chinese PLA Logistic Support Force, Nanchang, China; ^2^ Intensive Care Unit, Nanchang Hongdu Traditional Chinese Medicine Hospital, Nanchang, China; ^3^ Department of Critical Care Medicine, Hainan Hospital, Chinese PLA General Hospital, Sanya, China; ^4^ Heatstroke Treatment and Research Center of PLA, Sanya, China

**Keywords:** nafamostat mesylate, rats, heatstroke, proteomics, thrombosis

## Abstract

**Objective:**

To evaluate the impact of Nafamostat mesylate (NM) in improving survival outcomes among rats subjected to exertional heat stroke.

**Methods:**

This study involved a cohort of 45 specific pathogen-free (SPF) male Sprague Dawley (SD) rats. After successfully inducing exertional heat stroke, the rats were randomly divided into three groups: the Control group (Con, n = 15), the Exertional Heat Stroke group (EHS, n = 15), and the Nafamostat Mesylate group (NM, n = 15). A subset of ten rats from each group was selected for a 72-h survival analysis. Three hours following the successful establishment of the model, blood samples were collected under anesthesia for comprehensive analysis. This included routine hematological tests, coagulation assessments, and quantitative proteomics analysis, which were later validated using Parallel Reaction Monitoring (PRM). Additionally, tissue samples were harvested from the brain, heart, lung, kidney, liver, and duodenum of rats in each group for subsequent pathological examination.

**Results:**

The 72-h survival rate in the NM group was markedly higher than that observed in the EHS group. Pathological assessments indicated a notable reduction in thrombus formation within the brain, lungs, and liver in the NM group when compared to the EHS group. Furthermore, the NM group exhibited an elevated platelet count and a significant reduction in prothrombin time (PT) and activated partial thromboplastin time (APTT) relative to the EHS group. Proteomic profiling identified a total of 1,971 differentially expressed proteins, with 160 proteins being downregulated and 52 upregulated in the NM group as compared to the EHS group. PRM validation confirmed that the NM group significantly dampened the expression levels of key differential proteins, including ribosomal protein P2 (rpLP2), Histone 4c16 (H4c16), neutrophilic granule protein (NGP), and inositol monophosphatase 1 (Impa1), which are implicated in anti-inflammatory responses, suppression of immune-mediated thrombosis, and enhancement of cellular metabolism.

**Conclusion:**

NM mitigates coagulopathy, alleviates thrombus burden, and improves the 72-h survival rate in EHS rats through the modulation of differentially expressed proteins, specifically rpLP2, H4c16, NGP, and Impa1.

## 1 Introduction

The El Niño phenomenon, in conjunction with the greenhouse effect, has led to a yearly escalation in the prevalence of heatstroke, a condition that can be lethal and is marked by multi-organ failure (Lo et al., 2023). This condition stems from a disruption in the equilibrium between heat generation and heat loss, particularly in settings with high ambient temperatures and humidity, often presenting as hyperpyrexia and coma (Zhang et al., 2024). Exertional heat stroke (EHS), a subtype of heat stroke precipitated by intense physical activity, is commonly observed among individuals engaged in strenuous tasks, such as construction workers, military personnel, and athletes. EHS is typically more severe and is associated with higher mortality rates (Zhang et al., 2024). Individuals afflicted with EHS are prone to developing a coagulopathy known as heatstroke-induced coagulopathy (HIC), which significantly impacts negative prognostic outcomes (Bouchama et al., 2022). Research indicates that 40%–75% of EHS patients exhibit coagulation disorders, including thrombocytopenia and a deficiency in coagulation factors (Barletta et al., 2024). This consumptive coagulopathy markedly increases the risk of mortality among affected patients.

The pathogenesis of HIC is intricately associated with the inflammatory cytokine storm triggered by thermal stress, extensive vascular endothelial damage, and the swift consumption of coagulation factors (Abdullah et al., 2024). Anticoagulant therapy, which can ameliorate the depletion of coagulants, is recognized as a pivotal intervention in the management of HIC (Weber and Blakely, 1969). Traditional anticoagulants, such as heparin and low molecular weight heparin, have shown efficacy in the treatment of HIC; however, their inherent risk of inducing bleeding complications limits their widespread application in clinical practice (Yamakawa et al., 2020). Furthermore, considering that the pathogenesis of HIC transcends the realm of coagulation disorders, the concomitant use of robust anti-inflammatory therapies is considered to be of paramount importance within the holistic treatment approach.

NM, a synthetic serine protease inhibitor, manifests a diverse array of biological activities, with a pronounced emphasis on its anti-inflammatory and anticoagulant effects (Fujii and Hitomi, 1981). The short half-life of NM, at just 8 min, substantially diminishes the risk of bleeding associated with anticoagulant therapy (Aoyama et al., 1984). A wealth of research has substantiated NM’s efficacy in suppressing inflammatory responses (Issekutz et al., 1990), safeguarding endothelial cells (Kang et al., 2015), and moderating the activity of coagulation factors as well as the activation of the complement system (Matsuda et al., 1985; He et al., 2024). NM has become a cornerstone in anticoagulation protocols for extracorporeal life support (Sanfilippo et al., 2022). Clinical trials have demonstrated that NM significantly ameliorates survival rates in septic patients undergoing blood purification (Kamijo et al., 2020); yet, its efficacy in patients with HIC has not been thoroughly examined. In this vein, our study is poised to assess the prognostic impact of NM in a rat model of EHS and to delineate the precise mechanisms of NM intervention in EHS through advanced proteomic methodologies.

## 2 Materials and methods

### 2.1 Experimental animals and grouping

**FIGURE 1:**
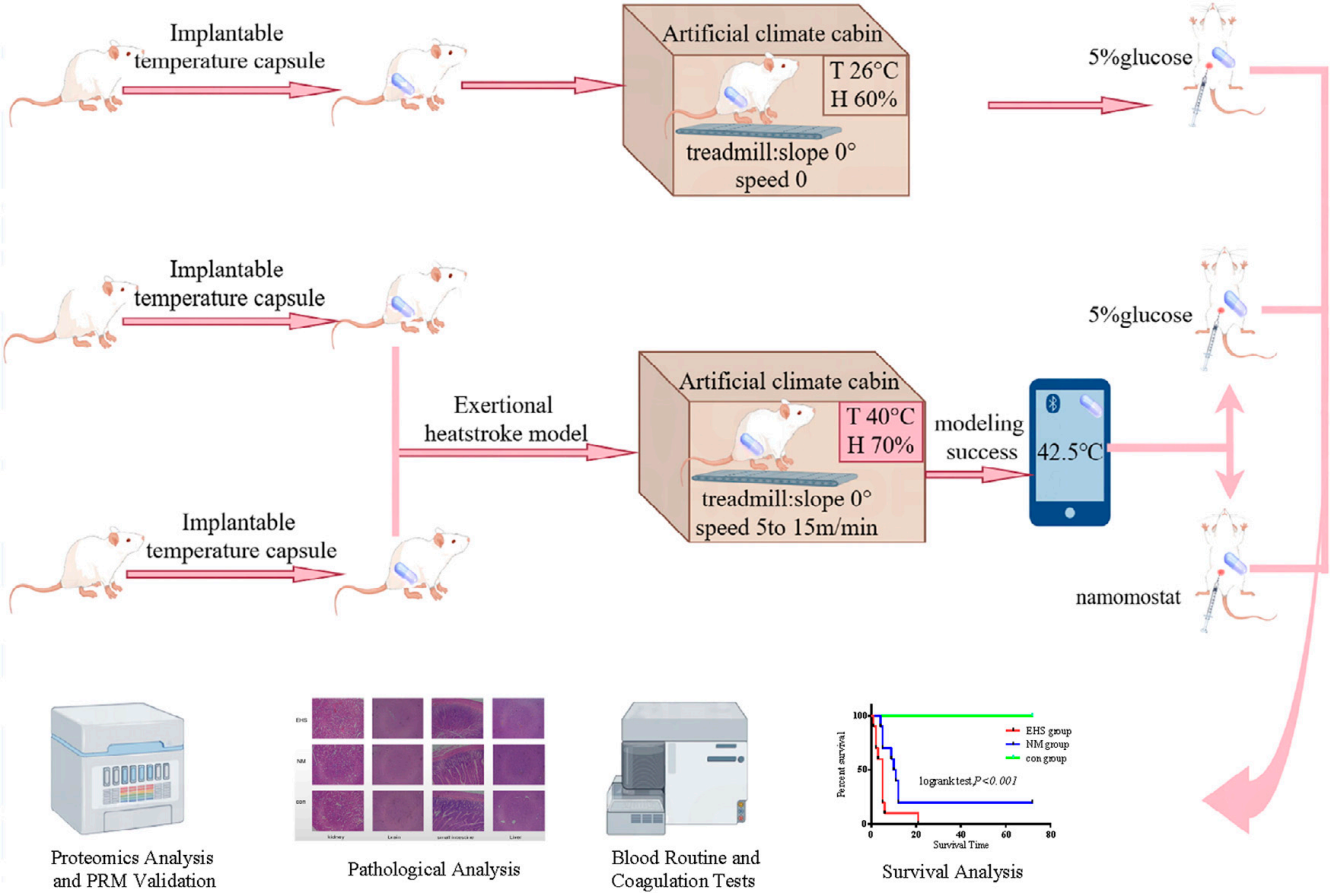
Study design and group allocation.

The experimental protocol was approved by the Ethics Committee of the 908th Hospital of Joint Logistic Support Force (Animal ethics approval number: 908yyLL2023073), and it followed the “replacement, reduction and refinement” principle (Flecknell, 2002). Forty-five specific pathogen-free (SPF) male Sprague Dawley (SD) rats were procured from Beijing Spibio Biotechnology Co., Ltd. (Animal Production License: SCXK [Jing] 2019-0010). The rats were permitted a 7-day acclimatization period in a controlled environment, with the temperature maintained at 25°C ± 1°C and relative humidity ranging from 40% to 50%, and they had *ad libitum* access to food and water. The study design involved the random assignment of 45 rats into three distinct experimental groups, each receiving a unique treatment protocol. The Control group (CON, n = 15) remained untreated. The Exertional Heat Stroke model group (EHS, n = 15) was administered an intraperitoneal injection of a 5% glucose solution at a dosage of 0.5 mg/kg. The Nafamostat mesylate group (NM, n = 15) received an intraperitoneal injection of NM solution at an equivalent volume (0.5 mg/mL). To induce the EHS model, the EHS and NM groups were housed in an artificial climate chamber set at a temperature of 40°C and a relative humidity of 70%, where they underwent treadmill exercise. For survival analysis, a random selection of ten rats from each group was performed. The experimental workflow is depicted in Figure 1.

### 2.2 Temperature telemetry capsule implantation

All rats underwent implantation of temperature telemetry capsules (Li et al., 2023). They were fasted for 24 h with limited water access prior to the procedure. Pre-surgical protocols involved weighing the rats and administering intraperitoneal anesthesia with pentobarbital at a dosage of 45 mg/kg. After sterilization, a 1–2 cm midline incision was made on the abdominal wall. The sterilized temperature telemetry capsule was then inserted into the abdominal cavity, and the incision was closed in layers, followed by sterilization of the surgical site. Postoperative monitoring of the rats was performed daily for 7 days to evaluate wound healing.

### 2.3 Establishment of EHS model

The rats were subjected to a 24-h fast prior to the commencement of the experimental protocol. The experimental chamber was maintained at an ambient temperature of 40°C with a relative humidity of 70%. Rats were positioned on a treadmill with an initial speed of 5 m per minute, which was incrementally adjusted to a steady pace of 15 m per minute. At the end of each lane, electrodes were positioned to deliver an electrical stimulus of 1 mA to motivate the rats to continue running. A rat was classified as fatigued if it ceased running for a period of 5 s despite receiving the electrical stimulus. Core body temperature was continuously monitored in real-time throughout the exercise. The exertional heat stroke model was considered successfully established when the rats exhibited signs of fatigue and their core temperature reached 42.5°C (Xv et al., 2024).

### 2.4 Comprehensive hematological analysis and coagulation profile assessment

Three hours following the successful establishment of the model, blood samples were rapidly collected in ethylenediaminetetraacetic acid (EDTA) tubes for comprehensive blood count analysis. Hemoglobin levels and platelet counts were determined using a fully automated hematology analyzer (Model Sysmex XT-2000iV, Japan). For assessment of coagulation profiles, blood was collected into sodium citrate anticoagulant tubes, maintaining a blood-to-citrate ratio of 9:1, and centrifuged at 3,000 revolutions per minute for 10 min at ambient temperature to obtain the serum. A fully automated coagulation analyzer (Model ACL TOP 700, United States) was employed to assess PT, APTT, thrombin time (TT), fibrinogen levels, antithrombin, D-dimer, and fibrin degradation products (FDP). Concurrently, blood samples from the control group were collected to evaluate the same parameters.

### 2.5 Histopathological examination

At the termination of the experiment, liver, lung, kidney, brain, and heart tissues were excised from all rat subjects and fixed in a 4% formaldehyde solution for 48 h. Following fixation, the tissues were processed through standard paraffin embedding protocols and sectioned to a thickness of approximately 5 μm. The sections were then subjected to deparaffinization by sequential immersion in xylene and a graded ethanol series. Subsequently, the sections were stained with hematoxylin for 5 min, followed by a rinse in distilled water. Differentiation was achieved using hydrochloric acid ethanol for 30 s, after which the sections were rehydrated in warm water for 5 min. Counterstaining was performed with eosin for an additional 5 min. The sections were then dehydrated and mounted for microscopic examination. Observations were conducted at a magnification of ×400, with five random fields selected per tissue section to evaluate thrombus formation. Determine the mean number of thrombi across these five fields of view to evaluate the overall thrombosis status.

### 2.6 Plasma proteomics analysis

Following the successful culmination of the modeling procedure, blood samples were procured from each experimental cohort 3 hours *post hoc*, with the objective of plasma isolation. Proteomic analyses of these plasma samples were executed utilizing Data Independent Acquisition (DIA) methodology. Initially, the plasma samples underwent desalination and concentration, which were essential precursors to the extraction and purification of the total protein content. Thereafter, the purified proteins were subjected to enzymatic digestion with a digestion buffer, yielding a collection of peptide samples. These peptides were subsequently interrogated via Liquid Chromatography-Tandem Mass Spectrometry (LC-DIA-MS/MS), thereby garnering DIA mass spectrometry data. The subsequent data analysis was conducted employing Spectronaut software, which facilitated the identification of peptides and proteins. This was complemented by a semi-quantitative analysis, employing label-free quantification strategies to discern proteins with differential expression patterns.

### 2.7 Parallel reaction monitoring

Differential proteins were identified using stringent criteria: a fold change (FC) exceeding 1.5 and a P-value below 0.05 were established as thresholds for significant upregulation, whereas an FC below the reciprocal of 1.5 (i.e., 1/1.5) and a P-value below 0.05 were set as thresholds for significant downregulation. To target the selected proteins for PRM, bioinformatics tools were leveraged to design specific peptides. High-resolution mass spectrometry was then applied to analyze these target peptides. Subsequently, PRM data were meticulously processed with Skyline software to quantitatively evaluate the differential expression levels of the target proteins within the experimental cohort.

### 2.8 Protein-protein interactions analysis by STRING database

The DEPs were meticulously curated based on stringent criteria: a pronounced upregulation exceeding 100-fold in the EHS cohort relative to the control cohort, and a downregulation of at least 0.5-fold in the NM cohort when contrasted with the EHS cohort. Post this rigorous selection, the protein names were queried in the STRING database, accessible via https://string-db.org, with the rat designated as the focal organism.

### 2.9 Data analysis

The measurement data were evaluated for normality using the Shapiro-Wilk test. For multi-group comparisons, data that conformed to a normal distribution were subjected to one-way ANOVA, with subsequent pairwise comparisons conducted using the LSD-t *post hoc* test. In scenarios where variances were not equal, Tamhane’s T2 *post hoc* test was implemented. For data that deviated from normality, the non-parametric Kruskal-Wallis one-way ANOVA was applied. The Chi-square test was utilized for the analysis of categorical data across multiple groups. When encountering expected frequencies of between one and five for at least two categories, or when any expected frequency was below one, Fisher’s exact test was employed. Survival data were analyzed using the Log-Rank test. For pairwise group comparisons, the significance level α′ was adjusted using the formula: α′ = 2α/K(K-1) = α/3 = 0.0167, with K representing the number of groups. A P-value below 0.0167 was regarded as statistically significant.

## 3 Results

### 3.1 NM enhances the 72-hour survival rate in EHS rats

**FIGURE 2:**
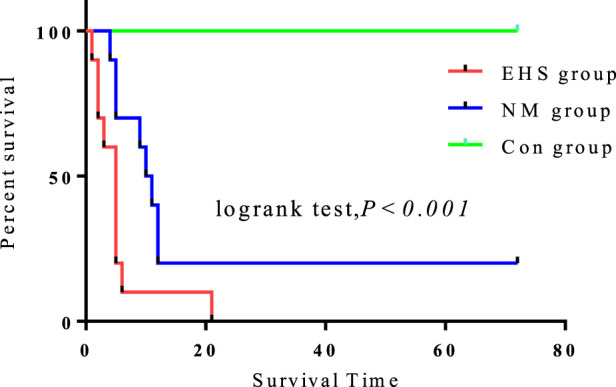
72-Hour survival analysis of rats by experimental group.

Compared to the control group, the 72-h survival rates for both the EHS and NM groups were significantly diminished (P < 0.05). Notably, the survival rate in the NM group was significantly higher, at 20%, compared to the absence of survivors in the EHS group. Despite no significant differences in pre-modeling body weight and core body temperature, duration of modeling, post-modeling dehydration rate, and time to temperature recovery between the NM and EHS groups (Table 1), the NM group’s survival rate was considerably higher than that of the EHS group (Figure 2).

**TABLE 1:** Comparison of baseline data between EHS and NM groups following statistical modeling.

Item	EHS (n = 5)	NM (n = 5)	p-value
Pre-modeling body weight (g)	204.90 ± 18.51	208.00 ± 19.17	0.717
Pre-modeling Tc (°C)	37.44 ± 0.30	37.74 ± 0.71	0.232
Modeling duration (min)	42.90 ± 17.77	46.80 ± 15.19	0.604
Dehydration ratio (%)	3.93 ± 1.58	4.15 ± 1.05	0.720
Temperature recovery time (min)	28.30 ± 7.12	28.80 ± 7.27	0.878

### 3.2 NM elevates platelet count and decreases PT in EHS rats

Relative to the control group, rats in both the EHS and NM groups demonstrated significantly extended PT and APTT, coupled with a pronounced elevation in red blood cell counts, hemoglobin levels, and hematocrit values, as well as a marked decrease in platelet counts (p < 0.05). In comparison to the EHS group, the NM group exhibited a significant elevation in platelet counts and a substantial reduction in PT (p < 0.05). No significant differences were noted in white blood cell counts, lymphocyte counts, fibrinogen levels, FDP, or antithrombin activity among the three groups (p > 0.05) (Table 2).

**TABLE 2:** Comparative analysis of hematological profiles and coagulation status among groups.

Item	Con (n = 5)	EHS (n = 5)	NM (n = 5)	F/U	*p*
White Blood Cell (×10^9^/L)	5.8 (5.6, 8.6)	11 (7.5, 15.6)	9.7 (6.6, 12.9)	3.142	0.208
Lymphocyte (×10^9^/L)	5.9 ± 1.8	6.8 ± 2.7	5.3 ± 2.7	0.485	0.627
Red Blood Cell (×10^12^/L)	7.2 ± 0.7	9.1 ± 0.9*	8.4 ± 0.8*	6.694	0.011
Hematocrit (%)	43 ± 2	56 ± 7*	53 ± 4*	9.498	0.003
Hemoglobin (g/L)	139 ± 8	175 ± 17*	172 ± 11*	13.393	0.001
Platelet Count (×10^9^/L)	1,311 ± 133	834 ± 111*	1,085 ± 230*^#^	8.06	0.018
PT (s)	10.5 ± 0.3	15.3 ± 1.7*	13.4 ± 1.5*^#^	16.2	<0.001
INR	0.9 ± 0.1	1.3 ± 0.1*	1.1 ± 0.1*^#^	16.079	<0.001
APTT (s)	18.5 (17.4, 20.6)	35.8 (26.8, 50.4) *	25.6 (22.4, 28.0) *	11.18	0.004
Fibrinogen (g/L)	1.07 (1.04, 1.09)	1.10 (1.0, 1.24)	1.16 (1.11, 1.33)	3.693	0.158
TT (s)	23.5 ± 2.2	25.6 ± 3.2	21.2 ± 4.8	1.887	0.195
D-dimer (μg/mL)	0.3 ± 0.02	0.4 ± 0.04*	0.3 ± 0.06	4.502	0.035
FDP (μg/mL)	0.5 ± 0.2	0.6 ± 0.1	0.5 ± 0.1	1.134	0.305
Antithrombin (%)	83 ± 1c4	84 ± 21	81 ± 12	0.037	0.964

PT, Prothrombin Time; INR, International Normalized Ratio; APTT, Activated Partial Thromboplastin Time; TT, Thrombin Time; FDP, Fibrin/Fibrinogen Degradation Products. * Compared to the Con group, P < 0.05; # Compared to the EHS group, P < 0.05.

### 3.3 NM alleviates microthrombosis in EHS rat organs

**FIGURE 3:**
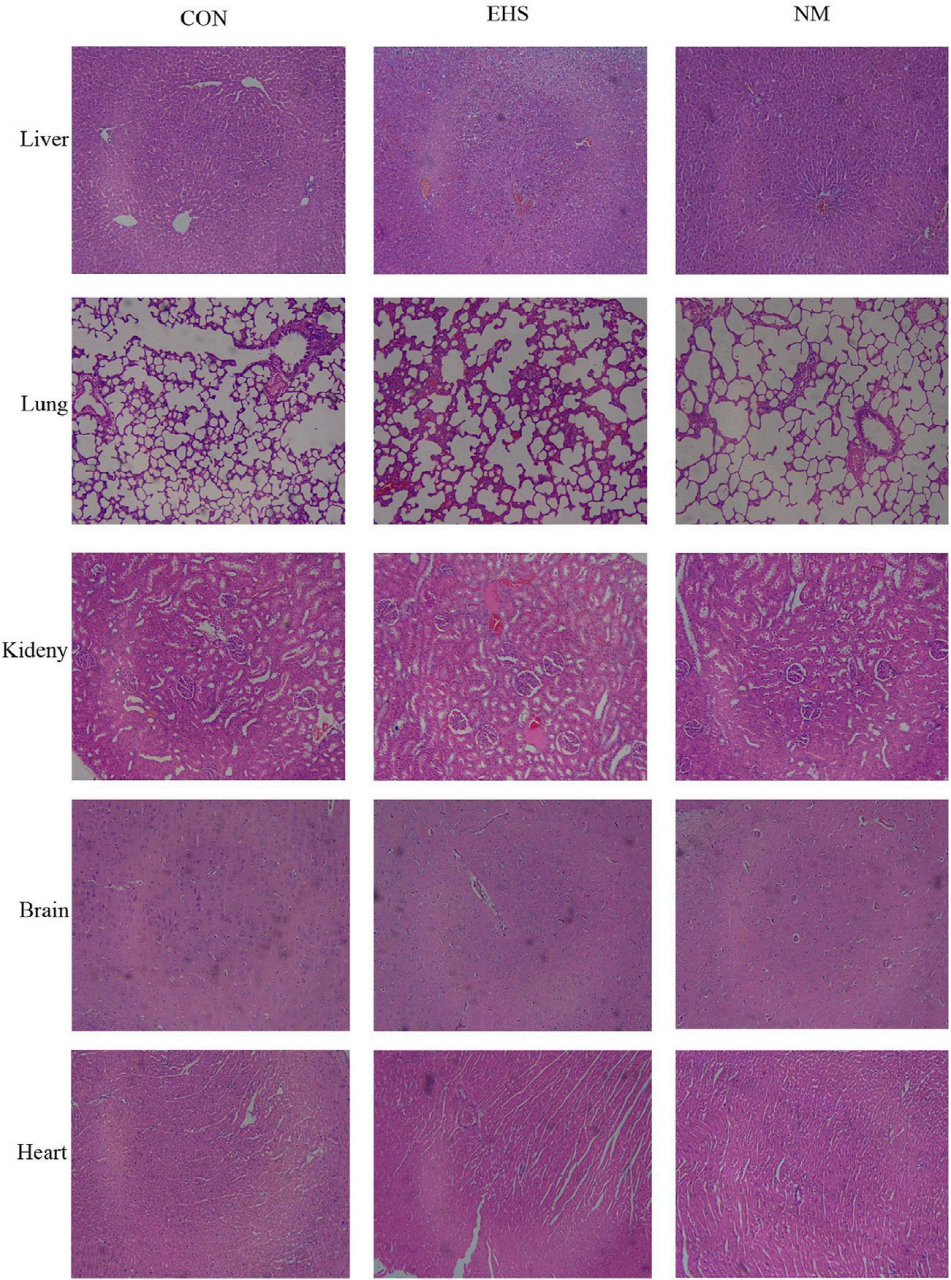
Histopathological alterations in various rat organs across experimental groups (Hematoxylin and Eosin stain, magnification ×400).

Histological assessments of tissues from the control group rats, including liver, lung, kidney, brain, and heart, demonstrated preserved tissue architecture without any significant pathological changes. Conversely, rats in the EHS group presented with pronounced pathological alterations. Specifically, hepatic tissues showed hepatocellular degeneration and necrosis, alongside sinusoidal dilation and congestion. Pulmonary tissues exhibited alveolar septal thickening, with evidence of alveolar collapse in some instances. Renal sections revealed tubular epithelial cell degeneration, intraluminal cellular debris, and interstitial edema affecting the renal tubules. Neuropathological examination of the brain identified neuronal degeneration and necrosis, coupled with a significant increase in glial cell proliferation. Additionally, there was a pervasive infiltration of inflammatory cells across these organs, with fibrin deposition and thrombosis observed in the microvasculature. Post-treatment with NM, a marked improvement in tissue disorganization was observed in the liver, lungs, kidneys, brain, and heart of the treated rats. This improvement was characterized by a significant reduction in inflammatory cell infiltration and a substantial decrease in thrombus burden, as illustrated in Figure 3.

As shown in Table 3, pathological examination revealed a statistically significant increase in thrombus count in the liver, lung, kidney, brain, and heart tissues of rats in both the EHS and NM groups compared to the control group (p < 0.05). Notably, rats in the NM group showed a significantly lower thrombus count in these tissues compared to the EHS group (p < 0.05). Among the organs examined, liver and kidney tissue sections exhibited a higher thrombus burden relative to the other tissues.

**TABLE 3:** Comparative analysis of thrombus distribution in organ tissue sections across groups.

Organ	CON (n = 5)	EHS (n = 5)	NM (n = 5)	*P*
Liver	0.0 ± 0.0	2.6 ± 0.8*	1.0 ± 0.2^*#^	<0.05
Lung	0.0 ± 0.0	2.0 ± 0.4*	1.2 ± 0.3^*#^	<0.05
Kidney	0.0 ± 0.0	1.2 ± 0.2*	0.2 ± 0.1^#^	<0.05
Brain	0.0 ± 0.0	1.0 ± 0.3*	0.5 ± 0.3^*#^	<0.05
Heart	0.0 ± 0.0	1.0 ± 0.6*	0.2 ± 0.1^#^	<0.05

*P < 0.05 compared with CON group, #*P* < 0.05 compared with EHS group.

### 3.4 Proteomic screening and validation of DEPs

**FIGURE 4:**
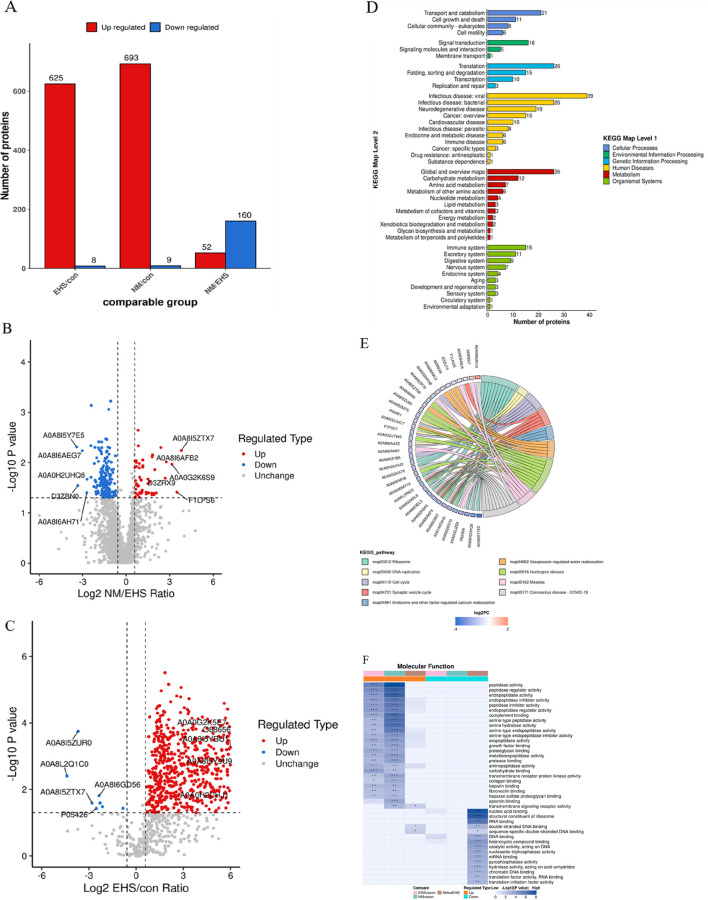
Comprehensive Proteomic Analysis. **(A)** Distribution of Differentially Expressed Proteins Across Groups. **(B)** (NM vs. EHS) This panel displays the volcano plot comparing protein expression between NM and EHS groups, with t-test P values indicated. The x-axis represents the Log_2_-transformed fold change ratio of differential protein expression, while the y-axis depicts the −Log_10_-transformed t-test P values. Proteins exhibiting significant upregulation are marked in red, those with significant downregulation in blue, and non-significant proteins in gray. **(C)** (EHS vs. CON): This panel displays the volcano plot comparing protein expression between EHS and CON groups, with t-test P values indicated. The x-axis represents the Log_2_-transformed fold change ratio of differential protein expression, while the y-axis depicts the −Log_10_-transformed t-test P values. Proteins exhibiting significant upregulation are marked in red, those with significant downregulation in blue, and non-significant proteins in gray. **(D)** KEGG Pathway Classification of Differential Proteins. This panel presents a KEGG pathway classification analysis for the differentially expressed proteins. The x-axis indicates the count of proteins within each category, and the y-axis delineates the secondary functional classifications under the overarching KEGG categories, which include metabolism, genetic information processing, environmental information processing, cellular processes, organismal systems, human diseases, and drug development. **(E)** KEGG Pathway Enrichment Analysis. A chord diagram visualizes the KEGG pathway enrichment analysis for differentially expressed proteins, highlighting the connections between significantly enriched KEGG pathways and their associated proteins. The enriched pathways are listed on the right side of the diagram, with proteins linked to these pathways arranged in descending order according to their Log_2_ fold change (Log2FC) values. **(F)** Functional Characterization of DEPs. This panel represents the functional characterization of proteins enriched across different comparison groups using color blocks that signify the degree of enrichment significance. A deep blue color indicates a high level of significance, while a blue-white gradient indicates lower significance. Significance levels are denoted by asterisks: * for P < 0.05, ** for P < 0.01, and *** for P < 0.001.

Enhanced by DIA technology, a meticulous proteomic profiling was executed, uncovering a total of 1,971 proteins across the plasma samples from each rat cohort. Subsequent comparative analyses revealed that, in juxtaposition to the control group, the EHS group manifested 376 proteins with differential expression, where 173 proteins were upregulated and 203 proteins were downregulated. A comparative analysis between the NM and EHS groups identified 212 proteins with differential expression, with 52 proteins upregulated and 160 downregulated. These proteins were predominantly linked to key biological processes, including inflammatory responses, coagulation and fibrinolysis, cellular signaling, and energy metabolism. KEGG pathway enrichment analysis highlighted a significant enrichment of these proteins in pathways pertinent to complement and coagulation cascades, cell adhesion molecules, ribosomal functions, and biodegradation metabolism (Figure 4).

The PRM validation process was conducted on a panel of 18 differentially expressed proteins (Table 4). The results revealed that Myh11 failed to meet the criteria for successful PRM validation. Additionally, the remaining 17 differential proteins have not been previously reported in the literature in relation to heatstroke.

**TABLE 4:** Validation of selected differential proteins via PRM.

Uniprot ID	Protein name	Gene	Average fold change (EHS vs. CON)	Trend (EHS vs. CON)	Average fold change (NM vs. EHS)	Trend (NM vs. EHS)	Previously associated with heatstroke
A0A0G2K6S9	Myosin-11	Myh11	10.1	No	0.5	No	No
A0A8I5YBU1	Rab GDP dissociation inhibitor	Gdi2	24.8	Yes	0.6	Yes	No
A0A8I6A721	Malate dehydrogenase	Mdh1	28.0	Yes	0.4	Yes	No
A0A8I6ABC9	Malate dehydrogenase	Mdh2	41.2	Yes	0.2	Yes	No
A0A8I6GMK2	14-3-3 protein beta/alpha	Ywhab	30.6	Yes	0.5	Yes	No
A0A8L2QPP4	F-actin-capping protein subunit alpha	Capza1	56.9	Yes	0.4	Yes	No
A0A8L2R623	AP complex subunit beta	Ap2b1	4.2	Yes	0.3	Yes	No
D3ZN03	Large ribosomal subunit protein P2	LOC100362751	111.9	Yes	0.1	Yes	No
D3ZY96	Neutrophilic granule protein	Ngp	171.1	Yes	0.4	Yes	No
F1M978	Inositol-1-monophosphatase	Impa1	297.6	Yes	0.3	Yes	No
G3V904	Phospholipase D family, member 4	Pld4	10.5	Yes	0.4	Yes	No
P05370	Glucose-6-phosphate 1-dehydrogenase	G6pdx	37.9	Yes	0.4	Yes	No
P42123	L-lactate dehydrogenase B chain	Ldhb	63.2	Yes	0.4	Yes	No
P46462	Transitional endoplasmic reticulum ATPase	Vcp	10.7	Yes	0.3	Yes	No
P62804	Histone H4	H4c16	242.4	Yes	0.4	Yes	No
P62828	GTP-binding nuclear protein Ran	Ran	25.0	Yes	0.4	Yes	No
P85973	Purine nucleoside phosphorylase	Pnp	57.2	Yes	0.5	Yes	No
Q91ZN1	Coronin-1A	Coro1a	54.5	Yes	0.5	Yes	No

### 3.5 Analysis of protein-protein interactions focused on four DEPs

**FIGURE 5:**
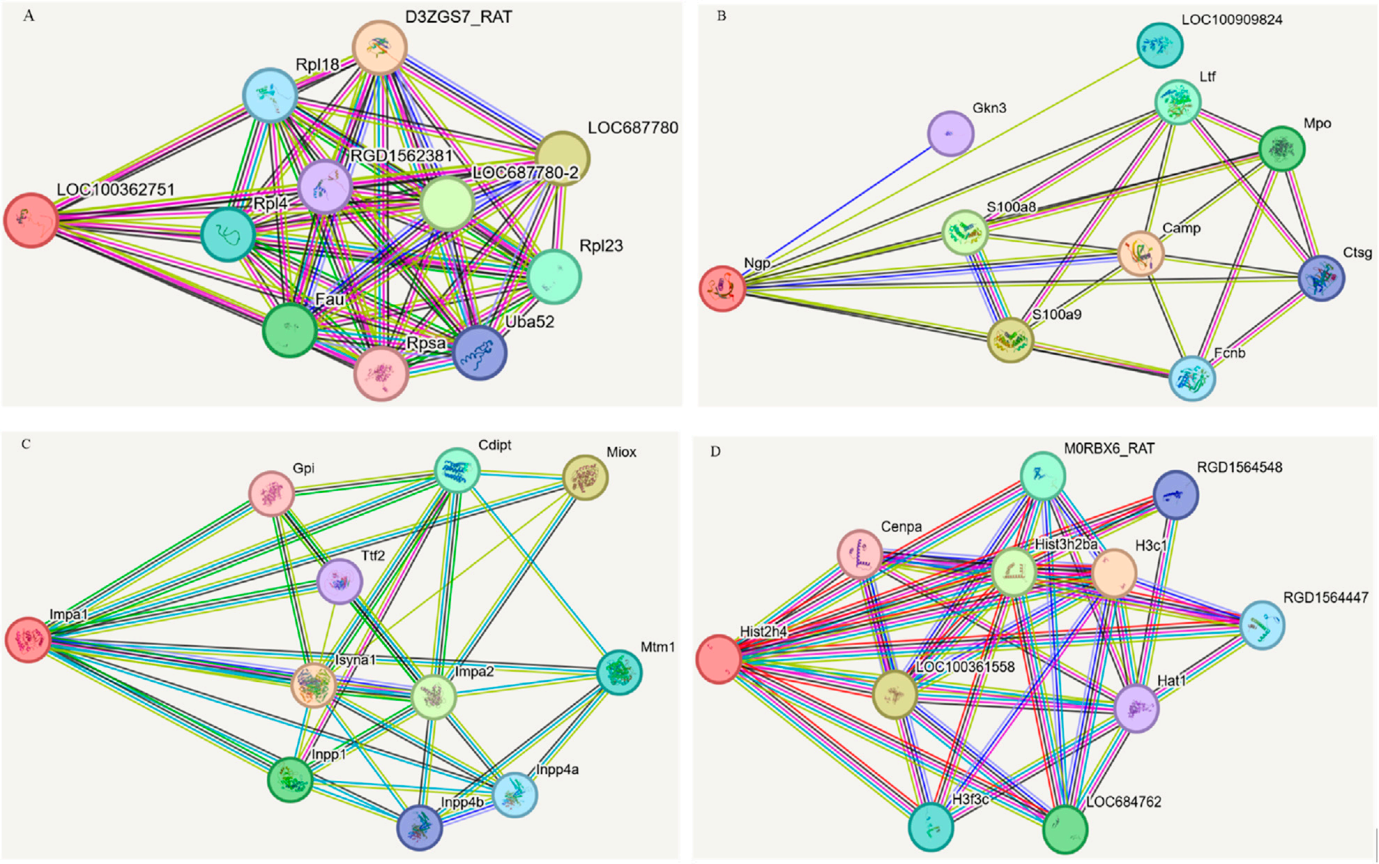
Protein-protein interactions focused on four DEPs. **(A)** LOC10036275. **(B)** Ngp. **(C)** Impa1. **(D)** His2h4 (H4c16).

Based on stringent selection criteria, four pivotal DEPs were identified as significant in the treatment of EHS with NM, including ribosomal protein P_2_ (rpLP2), Histone 4c16 (H4c16), neutrophilic granule protein (NGP) and inositol monophosphatase 1 (Impa1). Subsequently, these proteins were subjected to further analysis using the STRING database, with the rat species selected as the organism of interest. This approach facilitated the construction of protein-protein interaction (PPI) networks for each protein, as depicted in Figure 5.

Network analysis revealed that rpLP2 is localized in the nucleus, cytoplasm, and mitochondria, and is involved in cellular processes such as cytoplasmic translation and energy metabolism. The analysis identified several significantly enriched interaction clusters related to ribosomal components, signal transduction, and gene expression regulation, suggesting that this protein may interact with other proteins within these pathways. NGP, found in the lysosome, extracellular matrix, and cell membrane, plays a critical role in neutrophil aggregation, autocrine signaling, and response to chronic inflammation, with its primary molecular function being antioxidant activity. Network analysis highlighted two significantly enriched local clusters associated with bacterial killing within the neutrophil/granule lumen and metal chelation by antimicrobial peptides. The implicated signaling pathways include those related to antimicrobial peptides, neutrophil degranulation, immune response, and extracellular matrix metabolism. H4c16 is integral to the nucleus, cytoplasm, and mitochondria, and participates in essential biological processes such as cell cycle regulation, cell proliferation, signal transduction, immune response, metabolism, and apoptosis. Examination of H4c16 protein indicated enzymatic activity, binding activity (including DNA and protein binding), and transcription factor activity, emphasizing its role in gene regulation. Additionally, network analysis identified six significantly enriched interaction clusters associated with cell cycle control, signal transduction, and gene expression regulation. Impa1 is localized within the cytoplasmic small and large ribosomal subunits and plays a critical role in inositol metabolism, specifically in inositol monophosphate dephosphorylation and polyol degradation processes. Its primary molecular functions include inositol monophosphate 3-phosphatase and inositol 3,4-bisphosphate 4-phosphatase activities. The KEGG and Reactome signaling pathways associated with Impa1 pertain to inositol phosphate metabolism, the phosphatidylinositol signaling system, and phospholipid metabolism.

## 4 Discussion

This study introduces groundbreaking findings that NM, a synthetic serine protease inhibitor, significantly bolsters the 72-h survival rate in rats afflicted with EHS. Preceding studies have highlighted the role of NM in the efficacious management of sepsis-induced disseminated intravascular coagulation (DIC) in rats, attributed to its potent anticoagulant capabilities (Koshiyama et al., 1987). The EHS rat model was meticulously crafted under conditions of heightened temperature and humidity, with treadmill exercise, and core body temperature was meticulously tracked using telemetric capsules, thereby affirming the model’s reliability (Li et al., 2024). The complete mortality observed within 24 h post-modeling highlights the severity of EHS induced by the experimental conditions. Notably, NM achieved a 20% survival rate at 72 h in these severely affected EHS rats, a figure that, while modest, nonetheless demonstrates the therapeutic potential of NM in managing EHS. Moreover, the study’s data reveal that NM can reverse the thrombocytopenia and PT prolongation observed in heatstroke rats, and ameliorate microthrombus formation in the liver, kidneys, lungs, brain, and heart, suggesting that NM’s anticoagulant properties are pivotal in mitigating the coagulopathy associated with EHS.

Heatstroke-induced coagulopathy is predominantly characterized by “consumptive coagulopathy,” a condition marked by a significant depletion of coagulation factors and platelets due to the excessive activation of coagulation within the microvasculature (Weber and Blakely, 1969). This results in prolonged PT and APTT, as well as a decrease in platelet counts. As an anticoagulant, NM mitigates this pathological cascade through multiple mechanisms (He et al., 2024): it protects platelets from excessive thrombin activation, thereby curtailing aberrant platelet aggregation and consumption within the microvasculature, which in turn preserves higher levels of circulating platelets. Additionally, NM conserves coagulation factors by dampening the propagation of the coagulation cascade, thereby minimizing the wasteful consumption of these factors and maintaining their adequate levels, which results in the normalization of PT and APTT values. This multifaceted mode of action elucidates the initially paradoxical finding that NM therapy appears to reduce thrombus formation while concurrently elevating platelet counts and abbreviating PT and APTT.

To elucidate the proteomic alterations in EHS rats and to evaluate the effects of NM on these changes, we conducted a comprehensive proteomic analysis, supplemented by PRM validation. This approach revealed 17 DEPs that showed significant changes, none of which had been previously associated with the pathogenesis of heatstroke. Notably, the four DEPs with the most substantial alterations were rpLP2, H4c16, NGP and Impa1. The plasma concentrations of these proteins in EHS rats were strikingly elevated, reaching 110–300 times the levels observed in control rats. Post-NM intervention, these levels were markedly reduced to between 0.1 and 0.4 times the plasma levels found in EHS rats. These findings suggest a significant correlation between these four proteins and both the development of EHS and the therapeutic efficacy of NM.

RpLP2 is a pivotal component of the ribosomal P complex. Its phosphorylation enhances affinity for the elongation factor eEF-2, thereby augmenting the stability of the ribosomal complex and ensuring the fidelity of protein translation during biosynthesis (Yang et al., 2019). Prior research has documented significant upregulation of rpLP2 in systemic lupus erythematosus, severe hepatitis, nephritis, and certain malignancies, implying that dysregulated inflammatory responses are among its key pathogenic mechanisms (Ling et al., 2020; Artero-Castro et al., 2011). Although no existing literature directly supports this, the pronounced inflammatory response observed in heat stroke may potentially trigger the marked elevation of rpLP2 levels.

H4c16 is a histone 4 variant implicated in modulating chromatin architecture and gene expression (Poirier et al., 2006). Histone 4 typically exists as dimers and, in conjunction with histones 2A, 2B, and 3, constitutes the nucleosome. During sepsis, histones released from injured or dying neutrophils can induce hyperinflammation and widespread immunothrombosis, leading to ischemic organ damage (Li et al., 2022; Giglio et al., 2023). Our experimental findings indicate that significantly elevated histone H4 levels can serve as a biomarker for excessive immune thrombosis formation during heat stroke. Consistent with our results, previous studies have reported that serum histone levels can reflect the severity of heat stroke in dogs (Bruchim et al., 2017).

NGP has been reported to possess cysteine-type endopeptidase inhibitor activity and is involved in the defense against bacterial infections (Chen et al., 2019). During sepsis, NGP levels can significantly increase, reflecting the intensity of the inflammatory response (Mortaz et al., 2018; Malmström et al., 2014). IMPase 1 is an enzyme that dephosphorylates myo-inositol monophosphate to generate free myo-inositol. Myo-inositol is implicated in various biological processes, including intracellular signal transduction, calcium homeostasis regulation, osmotic pressure maintenance, and energy metabolism (Billcliff and Lowe, 2014). Elevated IMPase 1 level have been identified as a significant mechanism underlying bipolar disorder, and this enzyme has also been linked to the pathogenesis of several cancers, including cervical, renal, ovarian, colorectal, and breast cancers (Chen et al., 2023). This study is the first to demonstrate substantial increases in IMPase 1, rpLP2, and NGP levels during heat stroke, correlating with the acute metabolic perturbations observed in this condition.

NM, a synthetic broad-spectrum serine protease inhibitor, has demonstrated robust inhibitory activity against a spectrum of serine proteases, encompassing trypsin, elements of the complement system, the coagulation cascade, and the contact activation system (He et al., 2024). Its therapeutic potential is predominantly acknowledged in the treatment of both acute and chronic pancreatitis, as well as DIC (Kim et al., 2022). Moreover, NM potently inhibits the activity of transmembrane serine protease 2 (TMPRSS2), thereby impeding the binding of SARS-CoV-2 to angiotensin-converting enzyme 2 (ACE2), a pivotal mechanism underlying its therapeutic efficacy against COVID-19 (Hoffmann et al., 2020). This investigation marks the inaugural demonstration of NM’s efficacy in heat stroke treatment, revealing its capacity to notably diminish the elevated plasma concentrations of IMPase 1, rpLP2, H4c16, and NGP in heat-stressed rats. These findings intimate that NM may exert its therapeutic influence on heat stroke through mechanisms involving anti-inflammatory actions, suppression of immune-mediated thrombosis, and augmentation of cellular metabolism. The identification and validation of these four differentially expressed proteins through PRM provides several important insights into NM’s pharmacological mechanisms in heat stroke treatment. Most significantly, it establishes previously unrecognized molecular pathways through which NM might exert its protective effects beyond its well-documented anticoagulant properties. The modulation of IMPase 1, rpLP2, H4c16, and NGP suggests that NM’s therapeutic effect involves a complex interplay of multiple biological systems-including inflammation regulation, immune-mediated thrombosis, and cellular metabolism-rather than intervention in a single pathway. Our proteomics data demonstrate clear associations between NM treatment and alterations in these four proteins, providing potential molecular targets for future heat stroke therapies. However, the precise mechanistic relationships and causal pathways between NM administration and protein modulation require further investigation. Future studies focusing on functional validation through genetic manipulation or pharmacological inhibition of these specific targets will be essential to fully elucidate NM’s comprehensive mode of action in heat stroke treatment and potentially identify new applications for this promising therapeutic agent.

Despite these valuable insights, the study is not without limitations. First and foremost, the experimental findings are based on a rat model, which requires further validation through clinical trials. Moreover, the severity of the model used in this study, characterized by a 42.5°C temperature threshold leading to 100% mortality in the control group within 24 h, may introduce bias in the assessment of statistical significance. Additionally, while proteomic screening has identified a group of novel DEPs, the specific pathways through which NM modulates these DEPs remain to be elucidated. Lastly, the potential of these DEPs as biomarkers or novel therapeutic targets for heat stroke requires further empirical validation.

In summation, this study presents pioneering evidence that NM significantly bolsters the 72-h survival rate in EHS rats and attenuates the coagulopathy observed in these subjects. The underpinning mechanisms of these salutary effects are linked to the downregulation of plasma levels of IMPase 1, rpLP2, H4c16, and NGP.

## Data Availability

The data presented in the study are deposited in the ProteomeXchange Consortium (https://www.iprox.cn/page/project.html?id=IPX0011885000), accession number PXD063689.

## Abbreviations

APTT, activated partial thromboplastin time; DIA, Data Independent Acquisition; EHS, exertional heat stroke; FDP, fibrin degradation products; HIC, heatstroke-induced coagulopathy; Impa1, inositol monophosphatase 1; NGP, neutrophilic granule protein; NM, nafamostat mesylate; PPI, protein-protein interaction; PRM, parallel reaction monitoring; PT, prothrombin time; rpLP2, ribosomal protein P2; SPF, specific pathogen-free; TT, thrombin time.
